# Annotations

**Published:** 1891-02-14

**Authors:** 


### Annotations.

**The Pressman, the Parson, and the Physiologist.**

"In the spring a livelier iris changes on the burnish'd dove;
In the spring a young man's fancy lightly turns to thoughts of love."

Let no worshipper of the Laureate be angry if we see the physiology which not only underlies, but in this case at any rate, constitutes the poetry. The "livelier iris," of the "burnish'd dove" is due to the spring environment which stimulates physiological activity; and the young man's fancy which "lightly turns to thoughts of love," has its origin in a quickened vitality, which is also due to the healthful conditions of the spring-time. In these pages there is a constant effort to return to first principles; because first principles are the minted gold which is the fundamental condition of all physiological expenditure and activity. We are not among those who deny the existence and superiority of the soul; but neither are we among those who deny the influence and essentiality of the body. Since Lucretius there has been no physiological poet: and Lucretius himself had no very conspicuous merits either as a poet or as a physiologist. Nevertheless, life, human life, and all that it is and does, and all that is done round about it, and against it, and for it, might well furnish a theme for a poem as majestic as "Paradise Lost and as tragic as the story of Faust and Marguerite. Some man of genius will yet see the miraculous wonders of the human body, and sing the mystery of its marriage with the mind. In the meantime our purpose is humbler, and more immediately practical. There is health and life in the breath of the spring. Depression and death have been enshrouded in the winter's cold and fogs. Londoners and the inhabitants of other large towns must now be at a low ebb of vitality. The time is close upon us when we may all take a plunge into the healing and vitalising waters of the spring time. But it cannot be well done in London, or in Manchester, or in Glasgow, or in Leeds. In those places we firmly believe that the "iris" of the "burnish'd dove" does not become "livelier" in the spring time, but remains dull; and even the young man's fancy, though it may brighten up a little in the spring, does not turn to thoughts of love as his does whose blood is purified and fired by the breezes of the country, the mountain, and the sea. To be plain, we town people are all getting uncommonly flat; we can feel it in the morning paper; and even the sixpenny weekly lacks fire and "go." It is all a matter of physiology. We cannot help ourselves. Pure blood means high spirits, brilliant fancies, and noble thoughts. What a pity it is that every pressman and parson is not also a physiologist! If he were there might then be some chance of the early dawning of the true golden age.

**The Sun and the Doctor.**

"Where the sun does not go, there goes the doctor," says the Italian proverb. The proverb, however, is somewhat like a prophet, in that it has little honour in its own country. The sanitation of Italy, as statistics show, and as many travellers know to their cost, is infamous. It is probable that if Italy were as sunless as England it would rapidly degenerate into a pest-house. The fact that in spite of the abominations which meet you in every main street, and which make the exploration of the side streets and alleys impossible, Italy is nevertheless a highly salubrious country—this fact speaks volumes for the poison-destroying and health-giving potency of the sun. All sorts of diseases, from consumption downwards, are mitigated or cured by sunlight and pure air. This is a great commonplace: and so is this other, that food is the most important of all physics. But how to get all classes of the people, and especially the poor, to believe that life and health dwell in the sun's beams—that is a great difficulty. The indiscriminate opening of windows in all weathers and at all hours of the day and night is not a good thing: but it is a hundred times better than never opening them at all. If the inhabitants of large towns were really intelligent and instructed they would watch for the sun as the sailor's wife watches from her cottage on the hill-side for the return of her husband's ship; and when they saw it shining they would open every large and small window in the house to its utmost capacity until it went down again. There is every reason to believe that the germs of such diseases as scarlet fever, diphtheria, typhoid, and other such deadly enemies, are entirely destroyed by strong sunlight. Not only, however, has the sun the power of making germs die, but it is equally endowed with the potency of making men live. It is probable that almost every inhabitant of Great Britain could bring at least double the quantity of sunshine into the house during the year that usually enters. But the bringing in of the sun means, according to our Italian proverb, the keeping out of the doctor. It is not wise to keep the doctor out when he is really needed, but the doctor himself is the first to prescribe that all reasonable methods shall be adopted for the preservation and increase of health. The period of long days and much sunlight is close upon us again; let every man and woman make sure that not only themselves, but also their children and their servants shall have the fullest opportunities, during the spring and summer, of taking in unlimited quantities of the inexpensive, but life-giving sunshine.

**A House on Wheels.**

The measure now before Parliament, which proposes to deal with the children of those who live in houses on wheels, has drawn general attention to that most delightful of all methods of seeing the country and spending life in summer. A good deal of opposition has been manifested in various quarters against Mr. George Smith of Coalville's undoubtedly philanthropic purpose. We do not profess to have made any very special investigations into the question, but from a somewhat extended knowledge of houses on wheels, and their inhabitants, we cannot help thinking that Mr. George Smith has some right on his side. Our present object, however, is not to express an opinion on the measures now proposed to be converted into law, but to call the attention of readers to what seems to us one of the most supremely delightful of all methods of spending the summer and seeing one's native country or the civilized world. Of course you must "first catch your hare," or rather get your house on wheels built, and then, too, you must have a couple, or if you want to do it properly, three or four very first-rate horses. Here then is an initial outlay of four or five hundred pounds. Horse keep and men's wages will not cost less than four or five pounds a week. But these preliminaries allowed for, it is evident that a modest income and ample leisure can give a small but well selected party six or seven months every year of enjoyment that cannot be surpassed. Rural England is delightful beyond a poet's dreams. Its parks, its lanes, its woods, its streams, its downs, its dells, its corn, its cattle, and last, but not least, its people, furnish a series of ever-changing scenes and circumstances which might rouse the dullest to enthusiasm. Dr. Gordon Stables says he spends seven months in the year in hard work, and the other five on wheels, somewhat after the manner we have indicated. Happy man! The cares of life, we will warrant, sit lightly on his brow! Given leisure, moderate means, and a suitable party, neither Mr. R. L. Stevenson in the South Seas, nor Mr. Lawrence Oliphant in Thibet, could furnish us with anything half so enjoyable as a long, long summer spent in England, Scotland, Wales, and Ireland in a house on wheels.

